# Ozone in Medicine: A Few Points of Reflections

**DOI:** 10.3389/fphys.2022.842229

**Published:** 2022-02-23

**Authors:** Lamberto Re

**Affiliations:** Former Researcher Clinical Pharmacology Department, Marche Polytechnic University, Ancona, Italy

**Keywords:** oxygen, Nrf2, systems medicine, ozone, oxidative stress

## Abstract

Notwithstanding the use of ozone in medicine has become widespread in many countries of the world, its real pharmacological action remains not completely clarified. We know that other than its uses as disinfectant, well documented by the literature since the beginning of the past century, the more recent medical use of ozone in several pathologies as described by the international literature is still poor investigated. Furthermore, following its clinical uses with excellent clinical responses on several heterogeneous diseases and pain, it is now clear that the biological activity of this gas is mediated by graded responses to the mild oxidative stress induced after its application. Thus, the ancestral environment of our cells, whose energy production is strictly bind to oxygen burn, may be mediated by common defenses probably linked to the ubiquitous signaling pathway mediated by Nrf2. Moreover, after the first description of the oxidative stress in the 1970s and the discovery of Nrf2 as transcription factor in 1994, we could observe a rapid growth of the literatures regarding its function as master regulator of a myriad of cellular processes and its association to a multiple pattern of diseases including aging. In conclusion, to our opinion, the Systems Medicine approach could finally give to us the real key to better understand the wide reported efficacy of ozone treatment.

## Introduction

The subject of ozone and its uses in medicine is still a surprising and controversial topic of discussion.

The reason could be probably due to the association with the terms therapy or medicine, being not usual that a gas of its characteristic can find a rational location in this topic, almost to a first superficial approach. From a more general point of view, we could find similar problems in considering physical activity and/or nutritional habits as possible therapeutic resources in medicine, classifying them as real medical therapies.

We all know that this is not the case because, except for the food science, we can’t find any training course on the medical school programs regarding the practice of physical activities and or diet supplementation. Despite this, it is well known to all, as proven by numerous scientific literatures, that both resources, sport, and food intake, are real therapeutic remedies for many ailments, including diabetes, overweight and many other metabolic problems, either for prevention or for treatment.

Nevertheless regarding ozone, looking at the millions of treatments delivered practically in every country of the world, a deeper evaluation must be done in the aim to better characterize the possible mechanisms involved after adequate administration of this gas by a biochemical, physiological and even pharmacological points of view.

To my opinion, it is very sad to note that among the various psychological, physical, and other complementary approaches like “***dance***” recognized by one of the major world health institutions like NIH (NIH US, 2022), ozone is practically ignored.

To wide research it could be noted that there are some hints only on few Health Institutions like the DHA site as a complementary resource (DHA UAE, 2022), Australian TGA for dentistry (TGA AU, 2022), Cuban health institution (MSP Cuba, 2022), and perhaps other.

This fact could represent the beginning of a new era, where this gas starts to be considered not only toxic but useful in many pathological conditions so far orphan of adequate treatment, other than in reducing the damage of aging.

After many years of study and in the light of the most recent literature, I am even more convinced that ozone treatment could be better defined as a resource to support our body and in prevention rather than as a medical therapy. This does not mean that ozone treatment cannot be a fundamental complement to pharmacotherapy and surgery in supporting the conventional medicine protocols.

Indeed, in my opinion it can be a powerful aid both in favoring the drug action and in the reduction of the inevitable side effects.

Nevertheless, after more than one century, incredibly there are still many obstacles to its wider dissemination. Most of them absolutely without any scientific context and mainly related to the impossibility of breath it. Literally, it seems more a justification for not talking about it than a real impediment to its use through other routes of administration!

Since the beginning of its clinical use some of the main questions, so far unresolved, are:

“*how the medical doctor could define the best dose to use in relation to the general condition of the patient*?”

“*By what criteria can we formulate the safest and most effective doses to administer*”?

Before going into the specifics of this topic, I would like to recall the story that over the years has made the use of ozone more justified in the medical field and not only.

However, to my opinion, in the aim to better understand the true functional and physiological significance of ozone treatment, I believe that a deeper and serious approach will be more appropriate and urgent.

At the purpose and in the aim to characterize its supposed activity, we had to open our mind to different schemes being the Michaelis-Menten law not suitable to characterize the mechanisms of action of low oxidative stress by a pharmacological point of view.

## Biochemical Background and Pharmacological Aspects

The evolution of most mammals on our planet depends strictly on oxygen and its mitochondrial metabolism capable of allowing life in a hostile environment like the one that temporarily hosts us. It seems at least probable that the cells of organisms so dependent on this molecule have been equipped with control mechanisms capable of verifying and possibly correcting any anomaly both in the supply and in the metabolism of oxygen. A brief historical appeal makes us understand how science has officially decreed the importance of the molecular mechanisms underlying the control of oxygen level and to the oxidative stress for the human health. Most of them have been awarded at the highest level with the Nobel Prize in Medicine throughout the decades.

In 1931 Otto Warburg was awarded for discovering the basics of cellular respiration.

In 1938 it was Corneille Heymans who received this highest honor for having discovered the receptors located in the carotid arteries that regulate the rhythm of respiration according to the availability of oxygen.

Finally, more recently, and in my opinion in a context that shows more affinity with the mechanism of cellular respiration and oxidative stress, were awarded with the Nobel the colleagues Semenza, Ratcliffe and Kaelin, for their studies on the metabolic pathway that controls and regulates the cellular level of oxygen.

**FIGURE 1:**
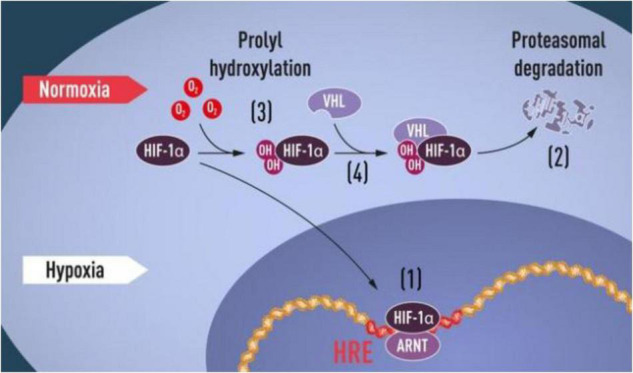
“HIF-1α Pathway”, Press release: The Nobel Prize in Physiology or Medicine 2019. NobelPrize.org. Nobel Prize Outreach AB 2022. Sat. 22 Jan 2022 (https://www.nobelprize.org/prizes/medicine/2019/press-release/).

It is clear that they discovered and described the fundamental adaptive machinery activated by a low level of oxygen which acts throughout gene modulation. Their studies have also favor the way for developing new strategies to combat anemia, cancer, and many other diseases.

As with HIF, the description of the metabolic pathway that controls another element of vital importance for humans, such as oxidative stress, began to appear in the scientific literature at the end of the last century.

It is up (Moi et al., 1994) to identify a transcription factor that will later be called Nrf2.

Successively, (Itoh et al., 1997) published the first work proposing Nrf2 as a transcription factor able to up-regulates hundreds and perhaps more genes involved in the cytoprotective response capable of restoring oxidative homeostasis and not only.

**FIGURE 2:**
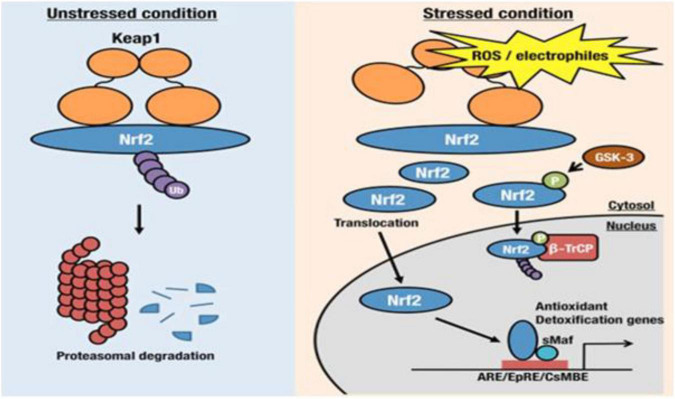
Keap1/Nrf2 pathway (https://www.sciencedirect.com/science/article/pii/S2468202016300067).

Under physiological conditions, that is, when the oxidative homeostasis of the cell is normal, Nrf2 is kept at a low level and, as with HIF-1α, it is degraded by the ubiquitin-proteasome system. Alternatively, in the case of ***mild oxidative stress***, ***environmental stimuli*** or ***pharmacological interactions***, the Nrf2 protein migrates toward the DNA segments of the ARE (***Antioxidant Response Elements***) area. This activation in turn favors the synthesis of proteins able to induce a regulation of the redox state and many other functions, in the aim to keep our cells healthy and protected from excess oxidative stress (Figure 2).

A wonderful lecture at the symposium “*Metabolism, life history and aging*” during the annual meeting of the Society for Comparative and Integrative Biology, 3 January 2010 to 7 January 2010, in Seattle, Washington, surprisingly anticipated the best definition of this metabolic pathway: ***Nrf2, Guardian of Health and Gatekeeper of Species Longevity*** (Lewis et al., 2010).

In light of the above, I think we can speak of a true “***therapy***” with the use of oxidative stress and ozone only in the case of problems related to herniated discs or other pathologies of the spine or of the musculoskeletal system as brilliantly described by Erario et al. (2021). All the other effects induced at the systemic level can be interpreted as the result of an adaptive response mediated at genetic level and activated by moderate oxidative stress or other agents.

Therefore, it appears to be intended more as a “***resource***” to maintain a healthy body by preventing apparently heterogeneous pathological conditions, such as ***immune, inflammatory, cardiovascular, neurological*,** and ***dermatological*,** as well as ***oncological*** conditions that share a common pathogenic basis.

The above concepts, far from being considered obsolete or out of date with the times, are surprisingly described also in prestigious scientific journals with a high impact factor (IF) by Cuadrado et al. (2018).

“***Life expectancy has almost doubled in the last century and specific diseases of aging are becoming prevalent.***

“*However, the pathological mechanisms underlying most of them are poorly understood and are treated more with symptomatic therapies rather than prevented correcting risk factors.*”

At the beginning of its uses the first hypotheses were that a brief oxidative stress could activate a multicellular mediated adaptive response throughout some metabolic pathways, mostly related to oxidative stress. Now, some recent discoveries introduced new elements that could better explain the positive effects of ozone treatments in medical conditions apparently so heterogenous but with the same pathogenetic mechanism.

By a pharmacology point of view new concepts and definitions following the “Systems Medicine” could be more coherent with the ozone action that could be defined better as Hormetic Stress (HS) or Epigenetic Therapy (ET). Indeed, as reported above, the mechanisms of action of ozone in mammalian, when used in adequate doses, is based on the fact that the brief and controlled oxidative stimulus leads to the formation of reactive oxygen species and lipid peroxides, which in turn act as second messengers. The apparently paradoxical concept that ozone could induce an antioxidant response capable of reversing transient oxidative stress is common in the animal and plant kingdom and it is supported by evidence of an increase in the level of antioxidant enzymes after a brief and adequate oxidative stimulus.

Today we know that cellular adaptation to oxidative stress is successfully modulated by an amazing intracellular mechanism: “*The Nrf2 Pathway.*” Although we should be happy with these new discoveries and new therapeutic resources, we realize that today there is still a lot of aversion and above all little credibility. To my opinion, the reasons of this poor consideration are not only ideological but, in some cases, based on real facts.

Cuadrado et al. (2018) in the excellent work cited above, described the complexity of a series of diseases called “***diseasome***” that share the same mechanism linked to nuclear transcription factor (erythroid-derived 2)-like 2 (Nrf2).

“*Interestingly, this network includes heterogeneous phenotypes such as* ***autoimmune, respiratory, digestive, cardiovascular, metabolic, and neurodegenerative diseases*,** *along with* ***cancer*** *and many other conditions*.”

Colleagues with some experience in the field of ozone will notice the similarity between the clinical results obtained with this gas with the same apparently heterogeneous diseases included in the group mentioned by Cuadrado. Nrf2 modulation is a common mechanism for all the diseases included in the same group.

**FIGURE 3:**
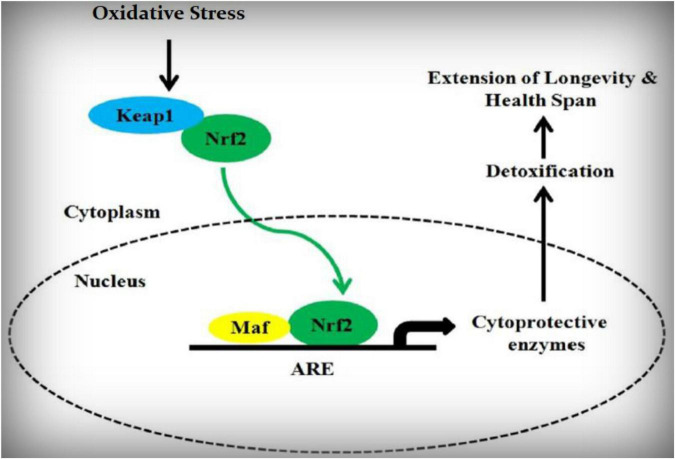
Nrf2 schematic pathway.

The scheme of its modulation is better shown in the following figure (Figure 3).

Furthermore, after the first description of oxidative stress in the second half of the 20th century and the discovery of Nrf2 as a transcription factor in 1994 (Moi et al., 1994), many other contributions have been proposed on the importance of Nrf2 as a regulator of a myriad of cellular processes.

Among these, some characterizing proliferation and differentiation (Kuosmanen et al., 2018), immune response (Hayes and Dinkova-Kostova, 2014), tissue repair (Braun et al., 2002), and many others related to cancer and cell survival (La Rojo de Vega et al., 2018). Other excellent contributions are that of Malhotra et al in which at least 1,055 Nrf2-dependent genes are proposed (Malhotra et al., 2010) and (Hybertson et al., 2011) were it is reported:

“*Nrf2 is known as the Master Regulator of the antioxidant response, modulating the expression of hundreds of genes, including not only the well-known antioxidant enzymes, but a host of seemingly disparate genes that control processes such as immune and inflammatory responses, tissue remodeling, and fibrosis, carcinogenesis and metastasis, and including cognitive and neurological dysfunctions in general.*”

What remained to be demonstrated was the evidence that, in addition to the pharmacological agents described in Cuadrado’s work, ozone could induce the activation of Nrf2 as well, thus explaining the surprising and multi-organ effects promoted by adequate doses of this gas.

To date we can find at least **57,392** papers in PubMed that contain the term “*Nrf2*,” of which **45,091** with the keyword “*Oxidative Stress*,” **1,328** with the term “*Ozone*” and **100** with “*Ozone Therapy.*”

The above numbers definitely demonstrate that moderate oxidative stress, and ozone in particular, is able to modulate the Nrf2 activity.

The first two articles that have demonstrated a direct effect of ozone on the Nrf2 metabolic pathway in humans are by Sagai and Bocci (2011) and confirmed *in vivo* by Re et al. (2014).

## Physiological Modulation

By a physiological and pharmacological point of view the difficult to assess randomized studies make us understand the problem.

One of the biggest problems in the interpretation and clinical evaluation of the treatments that act with an indirect mechanism compared to the usual drugs that have receptor action, is that of poor “***reproducibility***” and low “***reliability***” in relation to the doses to be used and the result obtained in terms of the patient’s clinical response.

In the case of ozone, whose action is consequent to the induction of moderate oxidative stress that in turn is capable to activate an adaptive antioxidant response through the metabolic pathway of Nrf2, the questions are many also taking into account the well know “*dark side*” of the Nrf2 which could promote negative interactions in cancer treatment (Wang et al., 2008).

For example, one of the most important is:

*what is the optimal dose of ozone, and therefore of oxidative stress, capable of initiating the activation of Nrf2 more effectively*?

Furthermore, what *is the level of “****upregulation****” of Nrf2 in patients with high oxidative stress*?

And *why is there no spontaneous activation of this metabolic pathway in patients with high oxidative stress*, or at least there are no data on its activation to date?

It is possible that, as in the case of the immune response, it is activated only in the presence of stimuli generated outside our body, that is, everything that comes from outside and is not recognized as “***self.***”

Could it be possible that even in the case of oxidative stress, only exogenous stress, which is foreign to the body and therefore comparable to that induced artificially by administering minimal doses of oxidizing agents such as ozone, is capable of activating the intracellular control system?

These questions and others, such as verifying whether identical doses of ozone are capable of activating Nrf2 to the same extent in subjects who have different degrees of oxidative stress, will be the primary and secondary end points that we will try to answer with a ongoing randomized clinical trial.

Our intention is to obtain essential information to optimize oxidative treatments and compare them with the usual direct antioxidant treatments induced by molecules such as glutathione, acetyl-cysteine and ascorbic acid.

As it is known, these substances have a “scavenger/purifying” action on free radicals and, therefore, a direct antioxidant effect.

In the case of oxidative treatments, such as ozone, the paradoxical antioxidant effect occurs indirectly through the activation of the Nrf2 metabolic pathway, which, as mentioned above, controls the oxidative state of the cell.

We will propose a first protocol in apparently healthy patients but with different levels of oxidative stress in which the level of the total oxidative state (TOS)/total antioxidant state (TAS) will be evaluated (Xiang et al., 2019) both before and after treatments with at least 3 doses of ozone (20, 35, and 50 μg/ml) using the systemic venous administration technique ex autohemotherapy (Project I).

## Clinical Applications

Following the more recent literatures regarding the concept of System Medicine we could include most of the diseases related to age and the elderly.

Regarding the doses, which must always be personalized, we must remember that the adaptive process is carried out with oxidative stimuli that must not exceed the toxicity threshold of the individual tissues where ozone is administered.

This is the reason that makes us understand the enormous variability of the doses used (from 5 μg/ml in cellulite to 50 μg/ml in intravenous systemic therapy) due to the different antioxidant capability of the individual tissues (low in skin and high in blood).

***This variability makes us better understand why it is impossible to use the airway due to the almost total absence of antioxidants on the surface of the pulmonary alveoli!***

**Integration and Supplementation represent** a very delicate field and too often it is interpreted and executed in a superficial and confusing way.

Sometimes it is essential to integrate oligo elements like selenium, manganese, copper or amino acids like arginine that are essential cofactors for a optimum antioxidant response at mitochondrial level.

Other important argument is the simultaneous administration of agents that directly promote the same effect as ozone, such as antioxidant agents like Vitamin C, glutathione and others.

In the first case, these cofactors should be administered before the ozone treatments, while in the second case only after having performed the treatment.

## Comments and Conclusion

The discovery that the Nrf2-dependent oxidative stress metabolic pathway can be activated by short and moderate oxidative stimuli opens up new perspectives, especially in consideration of the actual lifespan.

In fact, aging itself is a process resulting from the deterioration or imbalance of several factors, including the homeostasis of antioxidant/pro-oxidant processes that lead to an increase in reactive oxygen species (ROS) and multi-organ damage, especially in the elderly.

We fully agree with the idea that the complexity of the mechanisms underlying various diseases that share the same pathogenic causes as in the case of “***System Medicine***” cannot be studied with precision according to the existing protocols as in the case of organ (receptor) diseases.

On the contrary, we completely disagree with most of those who persist in thinking that ozone is always toxic by forgetting the simplest bases of both toxicology and human physiology with a total disregard for the advancement of knowledge!

The proposal to introduce a new approach for the clinical validation of drugs based on the study of the pathogenic mechanisms instead that of the single symptoms will be the most important challenge for the future of the new medicine, conceived precisely as “***System Medicine***” or “***Holistic Medicine.***”

Unfortunately, as happens all too often, it is observed with some regret that even in the Cuadrado’s prestigious work the ozone molecule is practically ignored. In fact, as part of his article, only electrophilic compounds and molecules (***bardoxolone*, *dimethyl fumarate***, etc.) capable of altering the Keap1/Nrf2 bond were cited as possible “***enhancers***” of Nrf2.

The fact that the Keap1/Nrf2 pathway may represent an “***auto-medicine***” for the cellular environment itself has stimulated the study of Nrf2 activators as possible drug candidates.

*New protocols based on oxidative therapies has recently proposed* (Re, 2020) *and it will be interesting compare its effects with the same produced by chemical species acting as direct activators on the same pathway* (Cuadrado et al., 2020).

I believe that the time has come for the scientific community to seriously reevaluate the use of ozone according to the most accredited protocols. I am sure that it can legitimately be considered as a strategic ally of the orthodox medicine, especially in the case of rare diseases that still lack adequate pharmacological treatments.

A key factor of no small importance is also represented by the almost absence of side effects.

Indeed, at the light of the millions of patients treated in the world in the last 40 years, its incidence is represented by a number after the decimal point preceded by at least 5 zeros!!!

Unfortunately, despite numerous clinical and scientific advances, the well-known respiratory toxicity of ozone remains an impediment to its use in the medical field. This still represents the main argument of those who continue to consider ozone only as a toxic molecule devoid of any possible therapeutic significance or at least useful in supporting vital functions!!

In my opinion, it is mandatory to introduce new methods and procedures to validate or not the benefits induced by this gas in multiple diseases, including aging, that share the same pathogenetic mechanism according to the concept of ***systems medicine***.

Unfortunately, too many divisions in the ozone therapy sector make this task difficult to achieve but we are sure that with the help and the supervision of supranational societies, scrupulously non-profit and without conflicts of interest, we will finally be able to start a serious and constructive path toward the full recognition of ozone uses in the medical field.

## Author Contributions

The author confirms being the sole contributor of this work and has approved it for publication.

## Conflict of Interest

The author declares that the research was conducted in the absence of any commercial or financial relationships that could be construed as a potential conflict of interest.

## Publisher’s Note

All claims expressed in this article are solely those of the authors and do not necessarily represent those of their affiliated organizations, or those of the publisher, the editors and the reviewers. Any product that may be evaluated in this article, or claim that may be made by its manufacturer, is not guaranteed or endorsed by the publisher.
